# Seasonal variation and interspecies dynamics among *Plasmodium falciparum* and *ovale* species in Bagamoyo, Tanzania

**DOI:** 10.1101/2025.03.12.25323778

**Published:** 2025-03-13

**Authors:** Kelly Carey-Ewend, Aidan Marten, Julia Muller, Editruda Ernest Peter, Melic Odas, Msolo Credo Dominick, Meredith Muller, Srijana Chhetri, Kano Amagai, Isaack Rutha, Fatuma Kisandu, Lusekelo Beka, Oksana Kharabora, Zachary R. Popkin-Hall, Jeffrey Bailey, Jessie K. Edwards, Emily W. Gower, Jonathan J. Juliano, Billy E. Ngasala, Jessica T. Lin

**Affiliations:** 1 Institute of Global Health and Infectious Diseases, University of North Carolina School of Medicine, Chapel Hill, NC 27514, USA; 2 Gillings School of Global Public Health, University of North Carolina, Chapel Hill, NC 27514, USA; 3 Department of Parasitology and Medical Entomology, Muhimbili University of Health and Allied Sciences, Dar es Salaam, PO Box 65001, Tanzania; 4 Department of Pathology and Laboratory Medicine, Brown University, Providence, RI 02912, USA; 5 Department of Microbiology and Immunology, University of North Carolina, Chapel Hill, NC 27599, USA

**Keywords:** Malaria, Plasmodium falciparum, Plasmodium ovale, Molecular surveillance, Asymptomatic malaria, Interspecies interaction

## Abstract

**Background:**

Malaria control in sub-Saharan Africa is typically focused on *Plasmodium falciparum* (*Pf*), but non-falciparum species like *P. ovale curtisi* (*Poc*) and *P. ovale wallikeri* (*Pow*) appear to be rising in prevalence, especially in East Africa.

**Methods:**

We conducted polymerase chain reaction (PCR)-based screening of 7,173 asymptomatic individuals over 5 years of age in coastal Tanzania from 2018–2022, employing real-time 18S rRNA PCR assays for *P. falciparum* and *P. ovale*, followed by *Poc*/*Pow* detection. *Plasmodium* positivity was compared across seasons and demographic groups, and interactions between species were analyzed via binomial regression.

**Results:**

*Pf* infection (prevalence 27.4%) was associated with younger age, male sex, and higher recent cumulative rainfall, whereas these associations were not apparent for *P. ovale* (*Po*, prevalence 11.5%). *Po* infections appeared to peak during months with lower *Pf* prevalence, especially during the long wet season, when *Po* mono-infections predominated and fewer *Pf*-*Po* co-infections were detected than expected by independent assortment. This apparent antagonism was reversed during the short wet season: *Pf*-*Po* co-infections were comparatively enriched despite low overall *Po* prevalence. In contrast, excess mixed *Poc*/*Pow* infections were detected across all seasons, composing 23% of the *Po*-positive isolates in which a specific *Po* species could be detected.

**Conclusions:**

The epidemiology of *P. ovale* species in coastal Tanzania suggests they are frequently present when *P. falciparum* recedes, but also co-infect the same hosts during the short wet season. Meanwhile, the individual *Poc* and *Pow* species often co-exist within individuals, perhaps due to co-transmission or concurrent relapse.

## Introduction

Four *Plasmodium* parasites - *P. falciparum (Pf)*, *P. malariae*, and *P. ovale* (*Po*) species *Po curtisi* (*Poc*) and *Po wallikeri* (*Pow*) - cause most malaria across sub-Saharan Africa, each in dynamic interplay as they co-circulate in the same communities. Prior observational studies, challenge infection experiments, and mathematical models have demonstrated variable propensity for multiple *Plasmodium* spp. to co-exist within individual hosts and vectors, and various intersecting mechanisms are thought to influence these patterns [1–11]. Excess detection of multi-species *Plasmodium* infections has been attributed to carriage within the same vectors, immune suppression, activation of relapses, and modulation of within-host niches (for example, anemia caused by *Pf* leading to reticulocytosis promoting *P. vivax*) [3,6,9,12–14]. Known heterogeneity in biting exposure and malaria risk may also lead to frequent co-infections among particular individuals in a given population [15]. Conversely, mutual suppression by within-host competition, immune protection, and differential distributions across vectors likely contribute to distinct detection of different *Plasmodia* across hosts [11,16,17].

Characterizing patterns of *Plasmodium* interaction is especially important given the changing landscape of malaria in sub-Saharan Africa. Transmission of *P. falciparum*, the primary cause of clinical malaria in the subcontinent, has declined in many regions and is generally much lower than the historical baseline [18]. Meanwhile, non-falciparum malaria spp., including *P. ovale* spp. infections, appear to be on the rise in some of the same regions where falciparum malaria has declined [6,18–21]. This recalls the persistence of *P. vivax* in regions where falciparum malaria has largely been eliminated, suggesting non-falciparum spp. may fill an ecological niche left by *P. falciparum* [22,23]. Since *P. ovale* spp infections are often low-density and subclinical, molecular screening is best suited to investigating these patterns.

We previously characterized asymptomatic *P. falciparum* carriage in Bagamoyo, Tanzania, showing considerable heterogeneity of transmission in an area where *P. falciparum* transmission has fallen but submicroscopic parasitemia remains prevalent [24]. We also reported substantial prevalence of *P. ovale* at our study site over the first three transmission seasons [25]. Here we present full data from more than three years of prospective cross-sectional PCR screening of roughly 7,000 individuals for *P. falciparum* and *P. ovale*, including *P. ovale* spp. identification, to investigate the epidemiology of co-circulating *Pf, Poc*, and *Pow* in East Africa.

## Methods

### Project TranSMIT

Participants aged 6 and older in the Bagamoyo district of Tanzania were recruited from schools and health clinics for cross-sectional malaria screening as part of the Transmission of Submicroscopic Malaria in Tanzania (TranSMIT) study [25,26]. Malaria transmission in this region occurs year-round, peaking during and after the long *masika* and short *vuli* wet seasons. Overall *P. falciparum* transmission has declined substantially compared to 20 years ago [27]. Recruited individuals reported no fever or antimalarial use in the last seven days. Following informed consent, dried blood spots (DBS) were collected alongside demographic survey data. This study was approved by institutional review boards at the University of North Carolina (ID 276606), Tanzania National Institute for Medical Research (NIMR/HQ/R.8a/Vol.IX/3150), Ifakara Health Institute (IHI/IRB/33–2018), and Muhimbili University of Health and Allied Sciences (MUHAS/DA.282/298/01/C).

### Molecular Screening

DNA was extracted from DBS using a chelex protocol prior to molecular amplification [28]. qPCR assays designed to amplify the *pf18S* rRNA gene in *P. falciparum* (*Pf*) [26] and the *po18S* rRNA gene in *P. ovale* (*Po*) spp. [28] were performed on-site in Bagamoyo using Sahara Hot Start PCR Master Mix (Chai Biotechnologies, Santa Clara, CA, USA) on portable Chai open qPCR machines, except for *Po* assays from samples collected in 2018–9 which were shipped to UNC for molecular detection. The *Pf* assay included a dilution series of positive control mock blood spots with known parasitemia (*Pf*) to enable estimation of parasite density, while the *Po* assay used *po18S* plasmid controls (MRA-180; BEI Resources). Positivity for *Pf* and unspecified *Po* spp. are defined in subsequent analyses as any amplification detected in either assay within 45 cycles. To eliminate false-positive *Po* detection due to cross reactivity in the qPCR assays, the *Po* detection threshold was adjusted down to ≤ Ct 42 in samples with measured *Pf* density over 100 parasites/μL [29].

Remaining DBS were shipped to the University of North Carolina at Chapel Hill, where extracted DNA was employed in qPCRs designed to amplify species-specific portions of the *po18S* rRNA gene between *P. ovale curtisi* (*Poc*) and *P. ovale wallikeri* (*Pow*) [30]. Determination of *Poc* and *Pow* positivity was based on the algorithm described in *Potlapalli* et al. [30] to avoid cross-reactive false-positive mixed infections.

### Statistical Analyses

Study data was stored and managed using REDCap (Research Electronic Data Capture) tools hosted at the University of North Carolina at Chapel Hill [31], analysis was performed using SAS software v9.04.01M7P080520 (SAS Institute Inc., Cary, NC, USA) with an α of 0.05, and data visualizations were produced in SAS or R v4.3.2 [32].

We visually investigated the relationship between age and *Pf*- and *Po*-positivity using locally-estimated scatterplot smoothing (LOESS) plots. We then used log-binomial regressions to estimate *Pf*/*Po* positivity by various functional forms of age and rainfall and compared them by Aikake Information Criterion (AIC). AIC and visual fit to LOESS curves led age to be coded categorically with breaks at 11yo and 15yo for downstream analyses. We similarly investigated the functional form of rainfall (mm/day, drawn from the CHIRPS database [33]), including lags of 6, 9, and 12 weeks (w) from date of rainfall to date of screening. This led to selection of restricted quadratic splines of 6w-lagged difference in average daily rainfall over the last month compared to the previous 3mo. The long wet season and short wet season are defined as March-May and October-December, respectively, with a 6w lag to screening date [34].

We modeled positivity for *Pf*, any *Po* species, *Poc*, and *Pow* as binary outcome variables for bivariate analyses examining their distributions by sex, age categories, season, and region. Region is defined as location of self-reported village of residence in the South or North of the district (Supplementary Figure 1). We reported proportions alongside Wald asymptotic confidence intervals and compared to demographic variables with Pearson’s *X*^2^. We performed an indirect standardization of wet season *Po* and *Pf* prevalence to the sex and age (in 5-year bins) population structure of Bagamoyo per the 2022 census [35]. We employed the Wilcoxon rank sum test to compare *po18S and pf18S* Ct values and the Kolmogorov-Smirnov test to evaluate differences in *Pf*/*Po* parasite density distributions between mixed and mono-infections. We calculated association between monthly *Po* and *Pf* prevalences using the Pearson correlation coefficient.

To assess adequacy of an interaction-free compartmental model to explain differential *Plasmodium* spp. detection, we fitted the raw data distributions to both a noninteracting distinct pathogens (NiDP) model (which assumes independent transmission of pathogens over the life course) and a multinomial model of independent assortment [36].

We then employed multivariate log-binomial regressions to estimate *Po* prevalence between individuals with and without *Pf* parasitemia. Interaction terms between season and *Pf* status were also included to allow estimated prevalence ratios to vary between seasons. We similarly modeled *Poc* prevalence by *Pow* status to estimate the association between positivity for either *P. ovale* species.

To account for bias in the *Poc*-*Pow* interaction caused by possible assay cross-reactivity and unknown *Po* spp. composition of some samples, we performed two sensitivity analyses using conservative mixed infection determination (requiring similar amplification of both species’ qPCR assays) and randomized assignment of unknown samples to mono-infections of either species (see Supplemental Materials).

## Results

### Epidemiology of co-endemic *P. falciparum* and *P. ovale*

**Figure 1.**
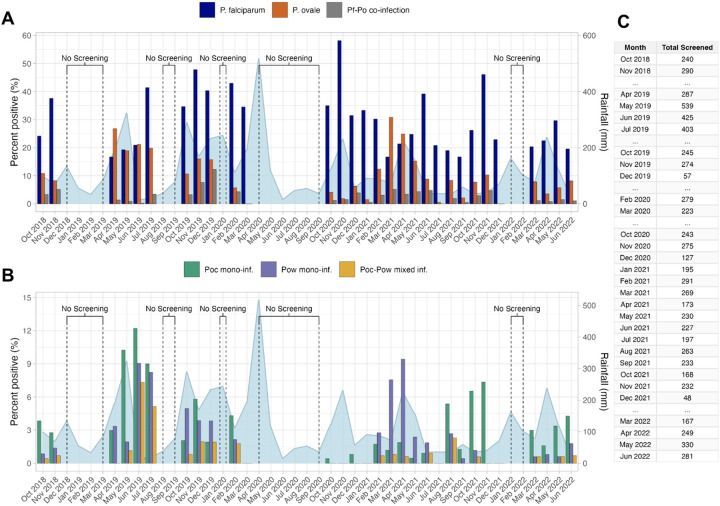
Cumulative monthly rainfall (mm, right) and prevalences (left) of *P. falciparum, P. ovale*, and *P. ovale-P. falciparum* co-infection positivity by month of screening in Bagamoyo, Tanzania, 2018–2022 (A), *P. ovale curtisi* (*Poc*) and *P ovale wallikeri* (*Pow*) positivity (B), and number of individuals screened per study month (C). Detectable *Poc* mono-infection (mono-inf.), *Pow* mono-inf., and mixed infection prevalences do not include *P. ovale*-positive samples in which the species-identification assay was not performed.

From October 2018 to June 2022, 7,173 asymptomatic individuals aged 6yrs and older (68% female, median age 18, IQR 11–30) underwent qPCR screening for *P. falciparum* (*Pf*) and *P. ovale* (*Po*) infection (Figure 1A, Supplementary Table 1). Screening was non-contiguous with no sampling during 15 of 45 months, including during the COVID-19 pandemic in 2020. Over the sampled period, 1,968 (27.4%, 95% CI: 26.4–28.5%) individuals were positive for *P. falciparum* and 827 (11.5%, 95% CI: 10.8–12.3%) individuals were positive for *P. ovale* spp. After performing an indirect standardization to the sex and age distribution of the Bagamoyo population, the estimated wet season prevalence of *P. falciparum and P. ovale spp*. among the population aged >5yrs was 30.6%, and 11.7%, respectively. Most infections were low-density: an estimated 71% of *Pf* and 83% of *Po* infections had <100 parasites/μL. Compared to when only one or the other was detected, *Pf*-*Po* spp. co-infections had slightly higher parasite densities (Supplementary Figure 2).

**Figure 2.**
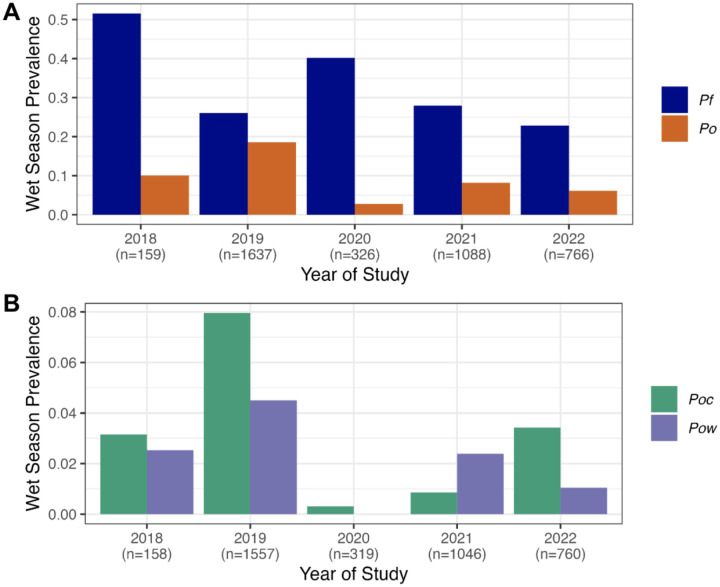
Prevalences of *P. falciparum* (*Pf*) and *P. ovale* (*Po*) (A) and detectable *P. ovale curtisi* (*Poc*) and *wallikeri* (*Pow*) (B) in the wet season (combined short and long) across study years. Y-axis range is reduced in (B) to show lower prevalence.

**Figure 3.**
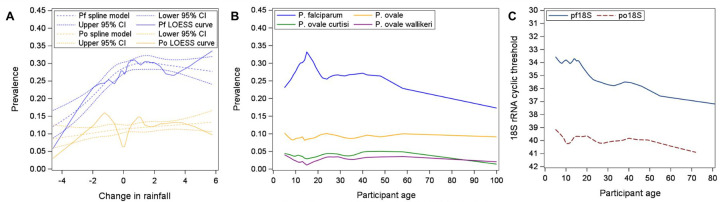
Prevalence of *P. falciparum* (*Pf*) and *P. ovale* by change in local rainfall (A) and participant age (B) among 7,460 screened individuals, and 18S rRNA polymerase chain reaction (PCR) Ct values by age among 826 and 1968 *Po-* and Pf-positive participants, respectively (C). Association of age is also shown by detectable *P. ovale curtisi* and *wallikeri* prevalence. Change in rainfall is coded as the difference in average rainfall (mm/day) between the preceding 1 month (mo) and the preceding 3mo, starting 6 weeks prior to screening. LOESS smoothed prevalences across the age and rainfall distributions are calculated using the 25% nearest observations; rainfall-prevalence relationships are also modeled using restricted quadratic spline models with breakpoints drawn at quartiles (alongside 95% confidence intervals [CI]).

*Pf* and *Po* prevalence varied seasonally and across study years, often in an oscillating manner (Figure 1). For example, *Pf* was most common in the short wet season (39% prevalence) and least in the long wet season (24% prevalence), while *Po* showed the opposite pattern (15% and 5% long and short wet season prevalence, respectively) (Table 1). Additionally, *P. ovale* prevalence appeared greater in periods with more modest *Pf* prevalence, and dropped when *Pf* transmission was greater, such that monthly *Pf* and *Po* prevalence were negatively correlated (r = −0.23, p = 0.21) (Figure 2A). While *Pf* prevalence increased after periods of more rainfall, we were unable to detect a similar trend for *Po* (Figure 3A).

Demographic characteristics associated with parasite carriage also differed between species. While *Pf* infections were more common among males and in older school-aged children (12–15yrs), *Po* infections were evenly distributed across sex and all age groups (Table 1, Figure 3B). These patterns persisted when examining *Po* infections without *Pf* co-infection. *Pf* parasite densities generally decreased with increasing age, while *Po* densities were low across all age groups (Figure 3C). Finally, while *Pf* was more prevalent in the northern part of the study area, *Po* spp. did not show any geographic structure (Table 1). *Pf* prevalence varied across different villages, ranging from 15–45%, but there was no association with *Po* prevalence (correlation of *Pf* and *Po* village-specific prevalence r = 0.04, p = 0.87) (Supplementary Figure 3).

### Seasonal dearth of *P. falciparum - P. ovale* spp. co-infection

**Figure 4.**
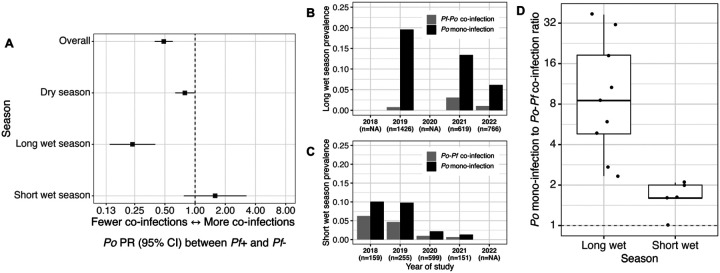
Seasonal variation in *P. falciparum* (*Pf*) detection among *P. ovale* (*Po*) carriers. Adjusted *Po* prevalence ratios (PR) by Pf co-infection status (A) and comparison of *Po* mono-infection and *Po-Pf* co-infection (B, C, D) by season. Log-binomial regressions compare *Po* prevalence between Pf-positive and negative individuals, overall and among different seasons, after adjustment for age, difference in rainfall over preceding 1 mo and 3mo, year of study, and season (in the overall estimate) (A). Po mono- and *Po-Pf* co-infection prevalences by year (B, C) and ratios by month (D) are split into participants screened 6 weeks after the long (March-May) and short (October-December) wet seasons. Mono-infection and co-infection refer only to presence of Po and *Pf*. Boxes in (D) reflect 1st, 2nd, and 3rd quartiles; whiskers extend to highest and lowest values within 1,5*interquartile range; data plotted on log scale.

Reflecting the contrasting epidemiology described above, the number of *Pf*-*Po* co-infections was lower than would be expected by independent assortment of the two *Plasmodia* in the population. Given a *Pf* prevalence of 27.4% and a *Po* prevalence of 11.5%, we would expect to detect 227 *Pf*-*Po* co-infections among 7,173 participants by independent assortment, but we observed 156 co-infections, 69% of the expected value. The data distribution also could not be explained by use of a noninteracting distinct pathogens (NiDP) model [36]. After adjusting for year of study, participant age, rainfall, and season, individuals positive for *P. falciparum* had 0.48 times (95% CI: 0.39–0.60; p < 0.0001) the probability of also being *Po*-positive compared to individuals without *Pf* infection. This antagonism was most pronounced in the long wet season (*Po* PR: 0.24, 95% CI: 0.14–0.40, p < 0.001), present in the dry season (*Po* PR: 0.79, 95% CI: 0.63–0.99, p = 0.04), and reversed in the short wet season (*Po* PR: 1.58, 95% CI: 0.77–3.25, p = 0.21) (Figure 4A). The ratio of *Po* mono-infections to *Pf-Po* co-infections in the short wet season were correspondingly low across study years, while long wet season ratios ranged significantly higher (Figure 4D).

### Epidemiology of co-endemic *P. ovale curtisi* and *P. ovale wallikeri*

Real-time PCR assays successfully detected *P. ovale curtisi* (*Poc*) or *P. ovale wallikeri* (*Pow*) in 376/631 (59.6%) of Po-positive samples tested (out of 827 total *Po*-positive samples). Among these 376, only *Poc* was detected in 189 (50.2%), only *Pow* was detected in 100 (26.6%), and both *Poc* and *Pow* were detected in 87 (23.1%) (Supplementary Table 2).

*Poc* and *Pow* parasitemia also varied over the course of the study in relation to each other and to *P. falciparum* transmission (Figure 1B). While *Poc* and *Pow* prevalences by study month were significantly correlated (Pearson correlation coefficient = 0.43, p=0.019) (Figure 2B), some alternating patterns were also present. For example, *Poc* predominated during the waning months of 2018, the latter half of 2021, and the first half of 2022, while *Pow* was the major *P. ovale* parasite found among those screened in the first half of 2021. During the period of highest *Po* spp. prevalence over the long wet season of 2019 (March-May), both *Poc* and *Pow* were detected, with a relative enrichment of mixed *Poc*-*Pow* co-infections in months following (June-July). Conversely, during the period of highest *Pf* prevalence over the short wet season spanning October 2020-January 2021, *P. ovale* prevalence was 1.5–6.3% and only *Poc* was detected.

Otherwise, the two *P. ovale* spp. showed generally similar patterns of detection by season, age, and sex (Table 2). *Poc* and *Pow* were both more likely to be detected in the long wet season compared to the dry season, and least prevalent in the short wet season. *Pf* was most common among adolescents, while *Pow* had the lowest prevalence among adolescents compared to children 6–11yrs and adults (p = 0.02) (Table 2, Figure 3B).

### Excess *P. ovale curtisi* and *P. ovale wallikeri* mixed infection

**Figure 5.**
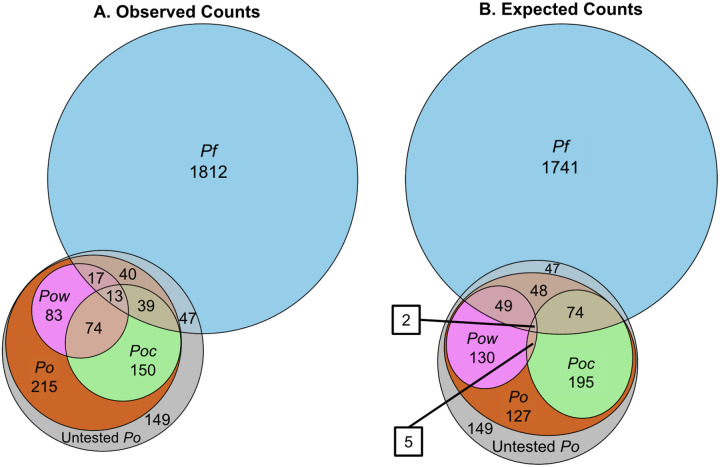
Distribution of observed (A) and expected (B) *P. falciparum* (*Pf*), *P. ovale* (*Po*), detectable *P. ovale curtisi* (*Poc*), and detectable *P. ovale wallikeri* (*Pow*) co-infections among 7173 participants. 196 Po-positive samples were not tested for species composition (Untested *Po*). Expected distributions were calculated assuming independent assortment of marginal positivity probabilities (excluding untested *Po* samples), not including untested samples: P(Pf-positive) = 0.274; P(Po-positive) = 0.090; P(Poc-positive) = 0.040; P(Pow-positive) = 0.027.

Unlike for *Pf* and *Po*, the observed prevalence of *Poc/Pow* mixed infections was higher than expected by independent assortment. *Poc* and *Pow* prevalences were 4.0% (289/6977) and 2.7% (187/6977), respectively, with 87 mixed infections observed in comparison to only 7 expected (Figure 5). Again, a NiDP model did not show better fit compared to an independent assortment model. Almost half of all *Pow* carriers had detectable *Poc* despite the relatively low prevalence of both organisms. This synergy persisted after adjusting for *Pf* infection status, age, year of study, and season: *Pow*-infected individuals had 15 times (95% CI: 13–18; p < 0.0001) the probability of also being *Poc-positive* compared to those without *Pow* infection.

To confirm that excess mixed *Poc/Pow* infection was not due to potential cross-reactivity in the 18S rRNA species-identification assays causing false-positivity of the minor species, we reduced the 87 mixed infections to 28 in which the *Poc* and *Pow* assays amplified within 3 cycles of each other; these represent mixed infections in which the minor species exhibits >10% the parasite density of the more prevalent species within the host. Using this conservative dataset, *Pow* carriers still have 3.7 times (95% CI: 3.1–4.5; p < 0.0001) the probability of having detectable *Poc* parasitemia compared to those without *Pow* infection (Supplemental materials).

## Discussion

This study provides a comparative portrait of co-endemic *P. falciparum* (*Pf*), *P. ovale curtisi* (*Poc*), and *P. ovale wallikeri* (*Pow*) within an East African community spanning nearly four years and 2,639 persons with asymptomatic *Plasmodium* carriage. We find disparate patterns of infection between *P. falciparum* and both *P. ovale* (*Po*) spp. and seasonal variation in their relative prevalence and degree of within-host overlap, suggesting underlying interspecies dynamics. Specifically, we observe a relative enrichment of *Pf*-*Po* co-infections during the short wet season, as would be expected if transmission relies on the same *Anopheles* vectors. However, this is outweighed by a relative paucity of *Pf*-*Po* co-infections during the rest of the year, with many more *Po* carriers without accompanying *Pf* co-infection during the long wet season. Conversely, among individual *P. ovale* species, we found frequent co-circulation in the same hosts, with marked enrichment of mixed *Poc*-*Pow* infections throughout the year.

We found few prior reports of varying prevalence of different malaria parasites in opposition to each other or with distinct seasonal patterns. *P. malariae* and *ovale* prevalence fell during the wet season when *falciparum* peaked in areas of Burkina Faso, Mozambique, and the Republic of the Congo, though these studies only examined individual years [37–39]. A weekly time series of malaria case data from mountainous areas in Peru showed brief spikes of *P. falciparum* detection that corresponded to troughs in *P. vivax* [40]. However, we could not find longitudinal studies that compared relative species prevalence in the long vs. short wet seasons in East Africa. Our findings may uniquely derive from the serial cross-sectional sampling across years as well as our focus on subclinical malaria.

If the observed seasonal differences in *Pf* vs. *Po* spp. prevalence reflect true infection dynamics in Bagamoyo, they could implicate seasonal differences in abundance of the vectors responsible for their transmission. While the three main malaria vectors in East Africa, *Anopheles gambiae sensu stricto*, *An. funestus s.s*., and *An. arabiensis* do fluctuate in their relative abundance, with some indication of seasonal alternating predominance [34,41,42] and variable *Pf* carriage [41–44], almost nothing is known about their relative roles as primary or secondary vectors for *P. ovale*. Sporozoite testing from mosquitoes collected in households in Burkina Faso in 2019–2020 revealed a higher *Po* prevalence in *An. funestus s.l*. vs. *Pf* predominance in *An. gambiae s.l*. Our team successfully infected colony-reared *An. gambiae s.s*. via direct skin feeding of *P. ovale*-infected individuals in Bagamoyo, with infectivity that seemed to surpass co-infecting *P. falciparum* in the same hosts [25], but we did not perform the same experiments in *An funestus*. If both *An. gambiae s.l*. (inclusive of *An. arabiensis*) and *An. funestus s.l*. are competent vectors for *Po*, predominance of *An. gambiae s.l*. at the onset of the long wet season and *An. funestus s.l*. near the season’s conclusion, as previously found in Tanzania [34], could lead to reduced *Pf*-*Po* co-infection despite substantial prevalence of both.

During the short wet season, detection of *Pf* and *Po* within the same hosts was higher compared to the other seasons. This finding is more aligned with the frequent *Pf*-*Po* co-infection observed in previous studies in East and Central Africa [4,5,7,8,19,21,45], involving both symptomatic and asymptomatic infections. Aside from abundance of both *Pf* and *Po* vectors in the short wet season, frequent co-infection in this period might derive from activation of *Po* relapses (from latent hypnozoites) by *Pf* super-infection, as is hypothesized to occur for *P. vivax* [14]. Further research into *Plasmodium* inter-species immunity, dynamics of ovale relapse, and vector surveillance for *P. ovale* spp. will be needed to elucidate mechanisms underlying the observed patterns. Worsening climate destabilization and attendant changes in seasonal cycles may add more complexity to deciphering these epidemiological patterns [46].

We successfully identified the specific *Po* species in 376 persons, finding that *P. ovale curtisi* (*Poc*) and *P. ovale wallikeri* (*Pow*) infection patterns largely mirror each other with frequent co-detection in the same hosts. Similar to prior surveys in East Africa [21,47,48], *Poc* slightly predominated, occurring at twice the prevalence among single *Po* spp infections, but *Poc* and *Pow* single-species infections exhibited similar associations with regards to age, sex, and season. Mixed *Poc*/*Pow* infections also appear common in prior small cross-sectional surveys [4,48,49]. This pattern might be attributable to co-transmission in vectors [30], relapse of one *Po* spp. triggered by super-infection of the other *Po* spp., or independent accumulation of both asymptomatic chronic infections over time. Frequent co-detection of *Poc* and *Pow* in the same hosts implies that control efforts, and changes to human and vector populations, may influence both parasite species in tandem.

Our study has multiple limitations. First, while it spans multiple years, screening was discontinuous and not consistent across years and seasons. This is partially addressed in multivariable regressions by adjusting for study year and rainfall. Second, demographic data for all participants were limited to age, sex, and village, and we crucially missed those <6 years of age. Developing immunity in young children may provide unique insights to infection patterns not evident in older age groups [42]. Third, even sensitive PCR assays likely miss low-density infections that circulate at or below their limit of detection [50]; longitudinal sampling probably represents the best strategy for detecting subclinical carriage. Fourth, failure of *Po* species-identification in 40% of positive samples limits our ability to characterize *Po* spp. infections with low parasite density, and assay cross-reactivity from field samples may bias towards detection of excess mixed *Poc*/*Pow* infections, though this was examined with robust sensitivity analyses. Finally, individuals with symptomatic malaria are not included in our survey. It is not known how often individuals with *P. ovale* spp. infection are symptomatic, but P. ovale continues to make up the minority of identified *Plasmodium* species among suspected malaria cases, ranging 0–15% prevalence among 10 Tanzanian regions sampled in 2021 [45]. Interspecies interactions and age-stratification likely manifest differently in symptomatic infections.

This survey of asymptomatic *Plasmodium* infections in East Africa shows seasonal disparities in patterns of infection between *Pf* and *Po* spp. alongside increased detection of *Poc* and *Pow* within the same hosts. These results indicate that *P. ovale* prevalence may be influenced by distinct factors from *P. falciparum* in the same community, including unique vector populations, human immune profiles, and relapse propensity. At the same time, *Poc* and *Pow* inhabit the same hosts without obvious differences in their epidemiology in older children and adults. Therefore, treating falciparum malaria and deploying interventions focused on groups at risk for *P. falciparum* are unlikely to adequately address *P. ovale* burden, while *P. ovale*-specific interventions have the potential to address both *Po* spp. in tandem. Further research that follows *P. ovale* spp. carriers over time, investigates the relationship between asymptomatic and symptomatic infections, uncovers each species’ relapse propensity and periodicity, and explores their clinical impact, will ensure that we pursue appropriate malaria control strategies (including potential treatment of hypnozoites) that do not neglect non-falciparum species.

## Supplementary Material

Supplement 1

Supplement 2

Supplement 3

Supplement 4

1

## Figures and Tables

**Table 1.** Unadjusted association of demographic characteristics to *P. falciparum* (*Pf*) and *P. ovale* (*Po*) positivity among 7,173 study participants. Region refers to the relative location of reported village of residence in the Bagamoyo district. Season refers to the long (March-May) and short (October-December) wet seasons incorporating a six week lag. Association of *Pf/Po* positivity with demographic variables is tested using Pearson’s *X*^2^.

Variable		N screened	*P. falciparum* positive N (%)	p-value	*P. ovale* spp. positive N (%)	p-value
Sex	Female	4853	1231 (25.4)		560 (11.5)	
	Male	2320	737 (31.8)	<0.0001	267 (11.5)	0.97
Region	North	3509	1092 (31.1)		411 (11.7)	
	South	2480	600 (24.2)	<0.0001	294 (11.9)	0.87
Season	Dry	3197	849 (26.6)		362 (11.3)	
	Long wet	2811	667 (23.7)		409 (14.6)	
	Short wet	1165	452 (38.8)	<0.0001	56 (4.8)	<0.0001
Age	6–11yrs	1411	372 (26.4)		151 (10.7)	
	12–15yrs	2018	603 (29.9)		217 (10.8)	
	>15yrs	3743	993 (26.5)	0.0149	459 (12.3)	0.13

**Table 2.** Unadjusted association of demographic characteristics to *P. ovale curtisi* (*Poc*) and *P. o. wallikeri* (*Pow*) positivity among 6,977 participants, and to *Poc* vs. *Pow* status among 287 *Po* spp. mono-infections (only one *Po* species detected). Association of *Po* spp. positivity (among all participants) and status (among mono-infections) with demographic variables is tested using Pearson’s *X*^2^.

Variable		N screened	*P. ovale curtisi* positive N (%)	p-value	*P. ovale wallikeri* positive N (%)	p-value	*P ovale curtisi* mono-infections (column % out of 187)	*P ovale wallikeri* mono-infection (column % out of 100)	p-value
Sex	Female	4721	185 (3.9)		134 (2.8)		124 (65.6)	73 (73.0)	
	Male	2256	91 (4.0)	0.82	53 (2.4)	0.24	65 (34.4)	27 (27.0)	0.20
Region	North	3412	135 (4.0)		94 (2.8)		91 (56.9)	50 (58.8)	
	South	2405	106 (4.4)	0.40	72 (3.0)	0.59	69 (43.1)	35 (41.2)	0.77
Season	Dry	3137	111 (3.5)		80 (2.6)		81 (42.9)	50 (50.0)	
	Long wet	2702	152 (5.6)		101 (3.7)		98 (51.9)	47 (47.0)	
	Short wet	1138	13 (1.1)	<0.0001	6 (0.5)	<0.0001	10 (5.3)	3 (3.0)	0.41
Age	6–11yrs	1378	52 (3.8)		40 (2.9)		36 (19.1)	24 (24.0)	
	12–15yrs	1970	71 (3.6)		36 (1.8)		60 (31.8)	25 (25.0)	
	>15yrs	3826	53 (4.2)	0.49	111 (3.1)	0.02	93 (49.2)	51 (51.0)	0.40

## Data Availability

De-identified datasets are provided as supplemental materials. Code for data processing, analysis, and visualization is available at https://github.com/IDEELResearch/TranSMIT_pf-po_epi.
