# Supplementary material for: Seasonal variation and interspecies dynamics among Plasmodium falciparum and ovale species in Bagamoyo, Tanzania
Source: medRxiv. 2025 Mar 13:2025.03.12.25323778. Preprint. [Version 1] doi: 10.1101/2025.03.12.25323778 (PMC11952601; doi:10.1101/2025.03.12.25323778)
Supplement: 1 [file NIHPP2025.03.12.25323778v1-supplement-1.pdf]

medRxiv preprint doi: https://doi.org/10.1101/2025.03.12.25323778; this version posted March 13, 2025. The copyright holder for this preprint (which was not certified by peer review) is the author/funder, who has granted medRxiv a license to display the preprint in perpetuity.
It is made available under a CC-BY 4.0 International license .

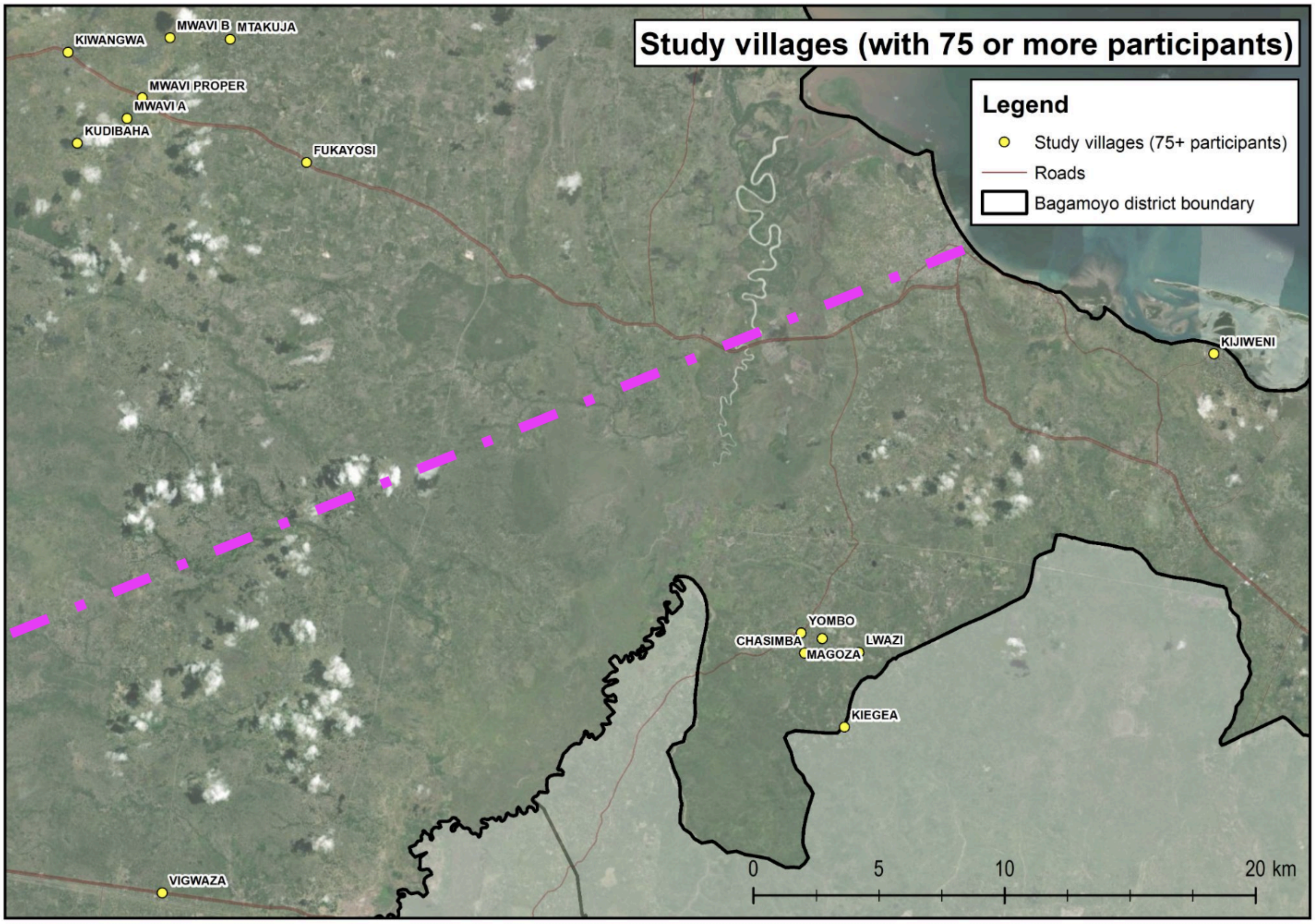

Supplementary Figure 1. Map of Bagamoyo district and villages with >75 participants screened. Dashed purple line shows the delineation between villages considered in the North and South of the district. Precise GPS coordinates for three villages in the North (Kwa Mkorea, Mwanamvuli, Zemba) and one in the south (Kisumbi) were not available. Reproduced from Rapp et al., *JID* 2024 [24].

medRxiv preprint doi: https://doi.org/10.1101/2025.03.12.25323778; this version posted March 13, 2025. The copyright holder for this preprint (which was not certified by peer review) is the author/funder, who has granted medRxiv a license to display the preprint in perpetuity.
It is made available under a CC-BY 4.0 International license .

Variable	Distribution: n (%, CI) or median (IQR)	Number missing data
P. ovale parasitemia	827 (11.5%, 10.8-12.3%) positive	1 (0.1%)
P. falciparum parasitemia	1968 (27.4%, 26.4-28.5%) positive	0 (0%)
po18S qPCR Ct	40.8 (38.8-42.1)	3/827 (0.4%)
pf18S qPCR Ct	35.8 (31.8-38.5)	2/1968 (0.1%)
Sex	4853 (67.7%, 66.6-68.7%) female	0 (0%)
Age (yrs)	18 (11-30)	1/7173 (0.01%)
Average Rainfall (mm/day in past month)	3.3 (1.5-6.3)	0 (0%)
Region	3509 (58.6%, 57.3-59.8) North	1184/7173 (17%)

Supplementary Table 1. Distribution of demographic and molecular screening variables among 7,173 participants screened from 2018-2022 in the Bagamoyo district of Tanzania. Region refers to localization of participant's self-reported village of residence to the North or South of the Bagamoyo district. *po18S/pf18S* = *Plasmodium ovale/falciparum* 18S rRNA subunit gene; qPCR Ct = quantitative polymerase chain reaction cyclic threshold; yrs = years; mm = millimeters; CI = 95% Wald asymptotic confidence interval; IQR = interquartile range.

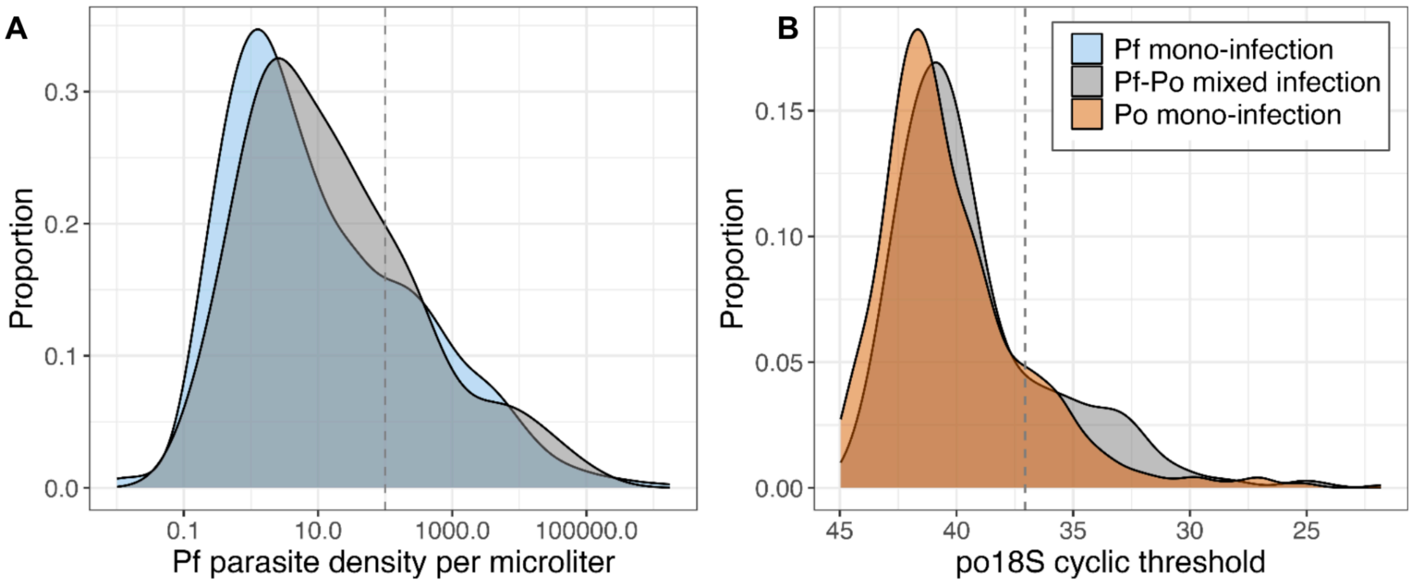

Supplementary Figure 2. Histograms of parasite density of *P. falciparum* (*Pf*)-positive samples (A) and *P. ovale* spp. (*Po*)-positive samples (B) among 1,812 *Pf* mono-infections, 671 *Po* mono-infections, and 156 *Pf-Po* mixed infections. *Pf* parasite density is calculated based on a standard dilution series amplified in the same *pf18S* PCR (polymerase chain reaction) assay as each sample, whereas *po18S* cyclic threshold (Ct) is presented as a proxy for *Po* parasite density because a *Po* standard series was not tested in each assay (low Ct corresponds to high density). Histogram bars are colored by presence or absence of the other *Plasmodium* parasite. Dashed vertical line depicts the submicroscopic threshold (100 parasites/μL); for B, this value was imputed from a *po18S* standard dilution series run separately. Parasite density distributions were different between mixed and mono-infections for both *Pf* and *Po* (p-values = 0.030 and 0.013, respectively).

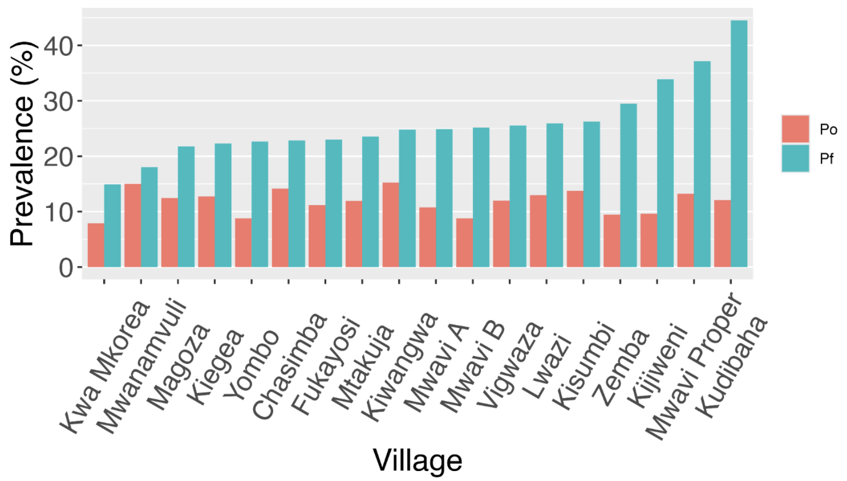

Supplementary Figure 3. *P. falciparum* (*Pf*) and *P. ovale* (*Po*) prevalences among the 18 villages with >75 participants screened. Villages are ordered by ascending *Pf* prevalence.

medRxiv preprint doi: https://doi.org/10.1101/2025.03.12.25323778; this version posted March 13, 2025. The copyright holder for this preprint (which was not certified by peer review) is the author/funder, who has granted medRxiv a license to display the preprint in perpetuity. It is made available under a CC-BY 4.0 International license .

Po species status	*Po*-negative	*Poc* mono-inf.	*Pow* mono-inf.	*Poc-Pow* mixed	Unknown species	No assay run
N	6346	189	100	87	255	196
%	88.5	2.6	1.4	1.2	3.6	2.7
% of tested *Po*+	N/A	30.0	15.8	13.8	40.4	N/A

Supplementary Table 2. Final species determination among 827 *P. ovale* (*Po*)-positive and 6,346 *Po*-negative participants. *Poc* mono-infection (mono-inf.) and *Pow* mono-infection reflect samples with amplification of the primer set for only one species or amplification of both species' primer sets at disparate levels that could be attributed to cross-reactivity in the assays. *Poc-Pow* mixed infections conversely reflect samples with shared amplification of primer sets for both species at sufficiently similar cyclic thresholds to represent simultaneous parasitemia with both *P. ovale* species (see *Potlapalli et al., PLOS NTD, 2023* [30]). The final reflects proportional species composition among 631 *Po*-positive samples in which the species-identification assays were performed. 255 *Po*-positive samples did not show amplification of either primer set, and 196 *Po*-positive participants lacked sufficient remaining dried blood isolate to run the assays.

medRxiv preprint doi: https://doi.org/10.1101/2025.03.12.25323778; this version posted March 13, 2025. The copyright holder for this preprint (which was not certified by peer review) is the author/funder, who has granted medRxiv a license to display the preprint in perpetuity.
It is made available under a CC-BY 4.0 International license .

Supplemental Materials

Sensitivity analyses employed to investigate excess of mixed infections

We employed a sensitivity analysis to ensure that the inflated probability of *Poc* and *Pow* co-detection within the same samples could not be reasonably explained by cross-reactivity between the 18S rRNA species-specific assays. Such cross-reactivity could present as false-positive low density amplification of the primer set for one species in relatively high density true infections of the other species [30]. While the algorithm we used for *Po* species assignment was designed to have 100% specificity for accurate calling of mixed infections, we performed a sensitivity analysis for potential *Po* spp. misclassification by limiting identification of mixed infections to samples in which the *Poc* and *Pow* qPCR assays amplified within 3 cycles of each other (representing mixed infections in which the minor species' parasite density represented over 10% the density of the more prevalent species within the host). Samples with more disparate amplification might indicate cross-reactivity and were reclassified as mono-infections of the predominant species in this conservative dataset, before

medRxiv preprint doi: https://doi.org/10.1101/2025.03.12.25323778; this version posted March 13, 2025. The copyright holder for this preprint
(which was not certified by peer review) is the author/funder, who has granted medRxiv a license to display the preprint in perpetuity.
It is made available under a CC-BY 4.0 International license .

repeating the above statistical analyses to estimate adjusted *Poc*-predominant infection prevalence ratios between individuals with *Pow*-predominant infections vs. individuals without.

An additional sensitivity analysis was performed to ensure that samples with unknown *P. ovale* species composition (*Po*-positive samples in which both species-specific qPCR assays failed) did not explain the observed excess of mixed infections, which would occur if mono-infections were more likely to go unidentified. Over 50% of detected mixed infections were in samples within the top tertile of overall *P. ovale* parasite density (which improves rate of successful species-identification), as compared to only 29% and 27% of *Poc* and *Pow* mono-infections, respectively (**Supplementary Figure 3A**). Maximum expected mixed infections would occur when the population prevalences of *Poc* and *Pow* are equal, so 10 simulations were performed randomizing the unidentified *P. ovale* samples to either *Poc* or *Pow* mono-infections at a ratio of 83:172, respectively, in order to equalize the final prevalences of *Poc* and *Pow*. Adjusted *Poc* prevalence ratios between *Pow*-positive and *Pow*-negative individuals were then calculated in each iteration, yielding 10 estimates.

These two sensitivity analyses were then performed in unison with both the conservative mixed infection identification scheme and randomization of unidentified samples to *Poc* and *Pow* mono-infections, yielding 10 prevalence ratio point estimates.

Using the conservative mixed infection classification approach, *Pow* carriers had 3.7 times (95% CI: 3.1-4.5; $p < 0.0001$) the probability of detectable *Poc* parasitemia compared to those without *Pow* infection. Among 10 simulations of randomizing the 255 unidentified *P. ovale* samples to *Poc* or *Pow* mono-infections, estimates of the adjusted *Poc* prevalence ratio between *Pow*+ and *Pow*- individuals varied from 5.4-5.7 and yielded significant p-values <0.0001 in all iterations. Use of both sensitivity analyses in tandem (conservative mixed infection classification and randomization of missing samples to mono-infections) showed that individuals with *Pow* parasitemia had 1.3-1.4 times the chance of having *Poc* compared to *Pow*-negative participants, with all p-values < 0.05. These indicate that the observed excess of *Poc*-*Pow* mixed infections in this cohort cannot be fully explained by increased assay cross-reactivity nor missing data bias from *P. ovale*-positive samples with unidentified species composition.

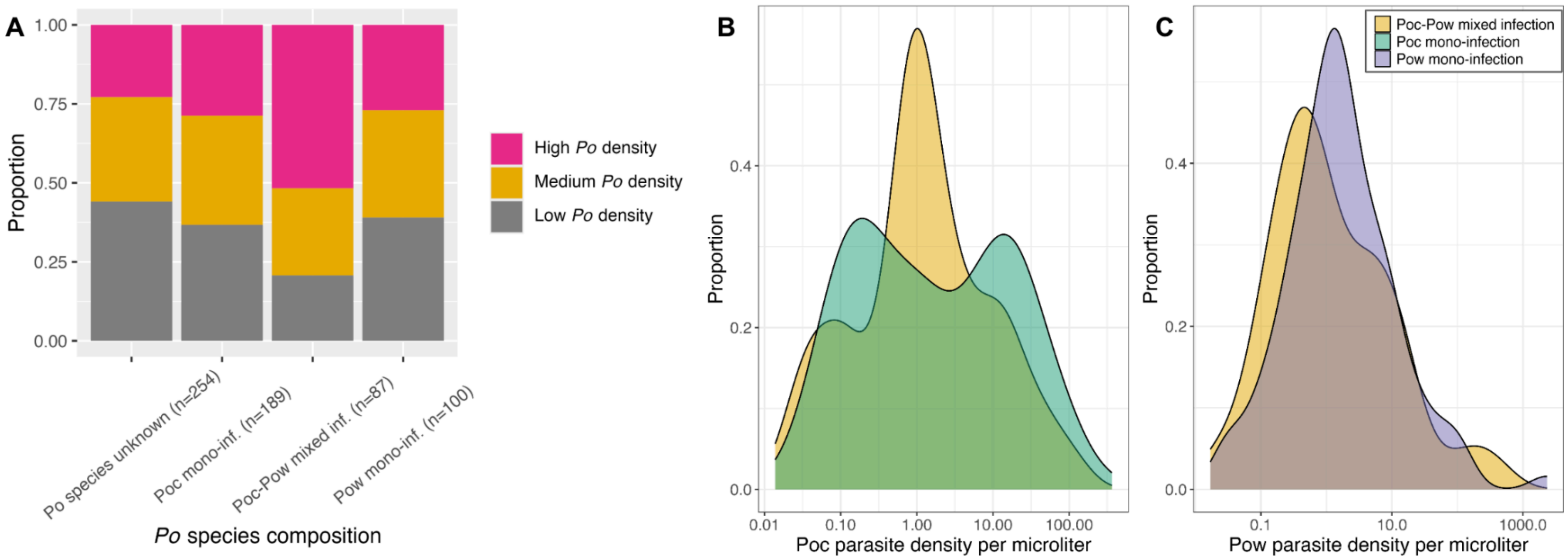

Supplemental Figure 3. Distribution of overall *P. ovale* (*Po*) parasite density (A), *P. ovale curtisi* (*Poc*) parasite density (B), and *P. ovale wallikeri* (*Pow*) parasite density (C) in *P. ovale curtisi* mono-infections (*Poc* mono-inf.), *P. ovale wallikeri* mono-infections (*Pow*), mixed infections, and infections with unknown *Po* species among 631 *Po*-positive samples which received both species-identification assays. *Po* density in (A) represents tertiles of all positive *P. ovale 18S rRNA* (*po18S*) gene quantitative polymerase chain reaction cyclic thresholds (Cts), with "high density" referring to the lowest tertile of Ct values. One *Po*-positive sample was found to be species unknown but lacked *po18S* Ct data and was excluded from this figure. Parasite densities in (B,C) are calculated based on a standard dilution series of plasmid controls amplified in the same assay as each sample. "Mono-infection" refers to an infection with only one *P. ovale* species detected, regardless of presence of other *Plasmodium* species.
